# Characterization of an *Enterococcus faecalis* bacteriophage SFQ1 as a potential therapeutic agent

**DOI:** 10.3389/fmicb.2023.1210319

**Published:** 2023-06-22

**Authors:** Fuqiang Song, Jun Sheng, Jishan Tan, Huajie Xie, Xiaoyu Wang, Wenqiong Guo

**Affiliations:** ^1^Department of Medical Laboratory, The General Hospital of Western Theater Command, Chengdu, China; ^2^Department of Orthopaedics, The General Hospital of Western Theater Command, Chengdu, China; ^3^Department of Clinical Pharmacy, The General Hospital of Western Theater Command, Chengdu, China; ^4^School of Nursing, Chengdu Medical College, Chengdu, China

**Keywords:** bacteriophage, *Enterococcus faecalis*, antibiotic resistance, phage therapy, vancomycin-resistant

## Abstract

*Enterococcus faecalis* is a well-established resident of the human gastrointestinal tract and is also a major cause of human infections. Unfortunately, therapeutic options for *E. faecalis* infections remain limited, particularly with the emergence of vancomycin-resistant strains in hospital settings. Consequently, there has been a renewed interest in phage therapy as an alternative to antibiotics. In this study, we have isolated a bacteriophage, vB_EfaS-SFQ1, from hospital sewage, which effectively infects *E. faecalis* strain EFS01. Phage SFQ1 is a siphovirus and exhibits a relatively broad host range. Furthermore, it has a short latent period of approximately 10 min and a large burst size of about 110 PFU/cell at a multiplicity of infection (MOI) of 0.01, and it could effectively disrupt the biofilms formed by *E. faecalis*. Thus, this study provides a detailed characterization of *E. faecalis* phage SFQ1, which has great potential for treating *E. faecalis* infections.

## Introduction

*Enterococci* are a group of low-GC Gram-positive cocci that comprise up to 60 different species (Cattoir, 2022). Among these species, *Enterococcus faecalis* and *Enterococcus faecium* are the two main species that have become a leading cause of human infections (Jabbari Shiadeh et al., 2019; Bright et al., 2020; Kakoullis et al., 2021). In recent years, the rise of vancomycin-resistant *Enterococcus* (VRE) has become a growing threat in hospital settings, and *E. faecalis* is the second most commonly isolated VRE species (Arias et al., 2011; Hayakawa et al., 2013). Moreover, *E. faecalis* could form biofilms that exist in the medical equipment, which makes the antibiotics ineffective against the bacteria in the biofilms (Venkateswaran et al., 2022). And the formation of biofilms makes it more difficult to cure infections (Ali and Neelakantan, 2022). Currently, the development of new antibacterial drugs is slow (Theuretzbacher et al., 2020). Consequently, there is an urgent need for novel therapeutic agents to combat multidrug-resistant bacterial infections (Gorski et al., 2016).

Phages, which are viruses that predate bacteria, can infect antibiotic-resistant bacteria, making them an attractive option for treating infections caused by multidrug-resistant bacteria (Dion et al., 2020; Strathdee et al., 2023). Phage therapy has already been successfully applied in various countries worldwide, including Belgium, Georgia, China, Germany, and the USA (Jault et al., 2018; Bao et al., 2020; Gelman et al., 2021; Leitner et al., 2021; Uyttebroek et al., 2022). Several clinical trials have been conducted to evaluate the safety and efficacy of phage therapy for various bacterial infections, including *Pseudomonas aeruginosa* infections in cystic fibrosis patients and *Staphylococcus aureus* infections in diabetic foot ulcers (Pires et al., 2020; Egido et al., 2021; Uyttebroek et al., 2022). While these studies have shown promising results, larger trials are needed to establish the safety and efficacy of phage therapy in a wider range of patients. On the other hand, more basic research is also needed to support clinical transitional research.

One of the key challenges facing phage therapy is the isolation of suitable phages for therapeutic use (Luong et al., 2020). The failure to isolate appropriate phages is common and can hinder rapid progress in phage therapy (Pires et al., 2015; Jault et al., 2018). Thus, isolation and characterization of phages are needed to perform phage therapy. In this study, we isolated a new lytic phage named vB_EfaS-SFQ1 from hospital sewage. Phage SFQ1 is a siphovirus and exhibits a relatively broad host range against *E. faecalis*, although it could not infect *E. faecium*. We provided a detailed biological and genomic description of phage SFQ1 and demonstrated its potential as a candidate for phage-based therapy against *E. faecalis* infections *in vitro*. Our findings contribute to the ongoing efforts to develop effective and safe phage-based therapies to combat multidrug-resistant bacterial infections.

## Materials and methods

### Strains and cultural conditions

Clinical strains of *E. faecalis* were isolated from the Department of Clinical Laboratory Medicine and subsequently preserved in our laboratory. To culture *E. faecalis*, brain heart infusion (BHI) medium was utilized and incubated aerobically with shaking at 37°C.

### Isolation of phage that infects *Enterococcus faecalis*

The bacteriophage was isolated from hospital sewage using the previously described method (Duerkop et al., 2016). Briefly, 2 mL of hospital sewage was centrifuged at 10,000 × g for 2 min, and the resulting supernatant was filtered through a 0.22 μm filter. The filtrate was mixed with log-phase bacteria EFS01 and cultured overnight with shaking at 37°C. The mixture was then centrifuged and filtered using a 0.22 μm aseptic filter. Next, 10 μL of the resulting supernatant was mixed with 100 μL of EFS01 host bacteria and added to 5 mL semi-solid BHI before being poured onto agar plates. The plates were then incubated overnight at 37°C until plaques formed. A single plaque was selected and purified by performing the plaque assay three times.

### Host range of SFQ1

To determine the host range of SFQ1, the double-layer agar method was conducted on a panel of clinically isolated *E. faecalis* and *E. faecium* strains (Table 1). The strains were grown in BHI medium and their susceptibility to lysis by SFQ1 was determined by the formation of clear plaques. The appearance of clear single plaques indicated sensitivity to the phage and the ability of SFQ1 to lyse the strain.

**Table 1:** The host range of phage SFQ1.

Strain	Origin	Phage sensitivity
*E. faecalis* EFS01	Sputum	+
*E. faecalis* EFS02	Sputum	+
*E. faecalis* EFS03	Sputum	−
*E. faecalis* EFS04	Sputum	+
*E. faecalis* EFS05	Urine	+
*E. faecalis* EFS06	Urine	−
*E. faecalis* EFS07	Urine	−
*E. faecalis* EFS08	Blood	+
*E. faecalis* EFS09	Blood	+
*E. faecalis* EFS10	Blood	−
*E. faecium* EFM01	Urine	−
*E. faecium* EFM02	Urine	−
*E. faecium* EFM03	Urine	−
*E. faecium* EFM04	Urine	−
*E. faecium* EFM05	Urine	−

“+” indicates that the strain is sensitive to phage SFQ1, and “−” indicates that phage SFQ1 did not form any plaque on the strain.

### Transmission electron microscopy

Phage SFQ1 morphology was characterized by transmission electron microscopy (TEM) following previously established protocols (Chatterjee et al., 2019). In brief, the filtered phage lysate was loaded onto a copper grid and incubated for 10 min. The grid was then negatively stained with 10 μL of 2% phosphotungstic acid for an additional 5 min. After air-drying, the samples were visualized using a transmission electron microscope operating at 80 kV.

### The optimal MOI of phage

To determine the optimal ratio of bacteriophages to bacteria that could produce the highest yield of progeny, the multiplicity of infection (MOI) needs to be determined. In this study, we investigated the optimal MOI for phage SFQ1 by mixing the phage and host bacteria at MOIs of 0.001, 0.01, 0.1, 1 and 10. The mixture was incubated at 37°C with shaking at 220 rpm for 5 h. The lysate was then filtered using a 0.45 μm filter, and the phage titer in each experiment was determined using the double-layer agar method. The experiment was repeated three times to ensure accuracy.

### One-step growth curve

The one-step phage growth process was conducted following the previously described method with some modifications. Initially, 5 mL of *E. faecalis* EFS01 in the exponential growth phase was mixed with phage SFQ1 at an MOI of 0.01. After 5 min of incubation, the mixtures were diluted by 200 times. The culture was further incubated at 37°C, and samples were collected at 10 min intervals. The phage titers in each sample were determined using the double-layer agar method.

### Stability of phage SFQ1

The stability of phage SFQ1 was evaluated using various assays. Firstly, pH sensitivity was tested by mixing 10 μL of concentrated phage stock solution (2 × 10^10^ PFU/mL) with 990 μL of BHI medium, adjusted with HCl or NaOH to pH values ranging from 2.0 to 13.0. The mixture was then incubated at 37°C for 60 min, and the phage titer was determined using the double agar layer method. The thermal stability of phage SFQ1 was tested by incubating the phage at temperatures ranging from 4°C to 70°C for 1 h, followed by determining the survival phage titer using the double-layer agar method. Additionally, the chloroform sensitivity assay was conducted by incubating phage SFQ1 (10^8^ PFU/mL) with varying ratios of chloroform (10%, 30%, 50%, 70%, 90%) at 37°C with shaking for 10 min. The mixture was then centrifuged, and the phage titer in the upper layer was calculated using the double-layer agar method.

### Phage genome extraction

Extraction of the phage SFQ1 genome was conducted following a previously described method (Han et al., 2022). Initially, DNase I and RNase A were added to the phage stocks to attain a final concentration of 1 μg/mL, which facilitated the removal of contaminated DNA and RNA. The mixture was then incubated at 37°C for 60 min. Subsequently, the phage genomic DNA was extracted using the phenol and chloroform method (Duerkop et al., 2016). The DNA was finally dissolved in sterile water and preserved at a temperature of −20°C until genome sequencing.

### Phage genome sequencing

The genome of phage SFQ1 was sequenced using the Illumina Hiseq 2500 platform. The reads were processed using Fastp to remove any errors and trimmed for quality control. Then, CLC software from QIAGEN (Germany) was applied to assemble the phage genome and annotated with RAST (Overbeek et al., 2014), an online tool that predicted the open reading frames (ORFs). Each ORF’s DNA and protein sequences were manually searched for homologs by BlastN and BlastP. Finally, the phage genome was visualized using the GraphPad Prism 8.0.2, and the sequence was deposited in NCBI under accession number OQ831052. The antibiotic resistance genes were predicted by the online prediction platform ResFinder^1^ and the virulence factors were predicted using the VirulenceFinder,^2^ respectively.

### Phylogenetic and comparative analysis of phage genome

To investigate the genetic relationships between phage SFQ1 and other *E. faecalis* phages, a phylogenetic tree was constructed using complete genome sequences of 21 phages, including SFQ1. The analysis was carried out with MEGA 7 software, which allowed for the assessment of similarities and differences in their genetic traits. Additionally, intergenomic similarities between SFQ1 and other *E. faecalis* phages were calculated using VIRIDIC (Moraru et al., 2020) to gain a deeper understanding of their relationships.

### Biofilm assays

The methodology for the biofilm assay was adapted from a previous study (Goodarzi et al., 2022). To establish a biofilm, fill each well of a 96-well plate with 100 μL of BHI medium containing an overnight culture of *E. faecalis*. The plates were incubated at 37°C for 24 h, 48 h, or 72 h to allow the biofilm to form. After removing the planktonic cells, 100 μL of phage with a titer of 10^8^ PFU/mL was added to each well, while control wells were added with 100 μL of SM buffer. Incubation was continued at 37°C for 6 h. Then, plates were washed with PBS and the biofilm biomass was stained with crystal violet for 15 min. To quantify the results, crystal violet was solubilized in 0.2 mL of 95% ethanol, and the OD_600_ was determined using a SpectraMax M3 multimode microplate reader. Each sample was examined in triplicate, and BHI medium alone served as the negative control.

### Statistical analysis

Each experiment was repeated three times to ensure the reliability of the results. The statistical analysis was carried out using GraphPad Prism 8.0.2, and student’s *t*-test was used where applicable. A *p*-value less than 0.05 was considered statistically significant.

## Results

### Isolation of *Enterococcus faecalis* bacteriophage SFQ1

**Figure 1:**
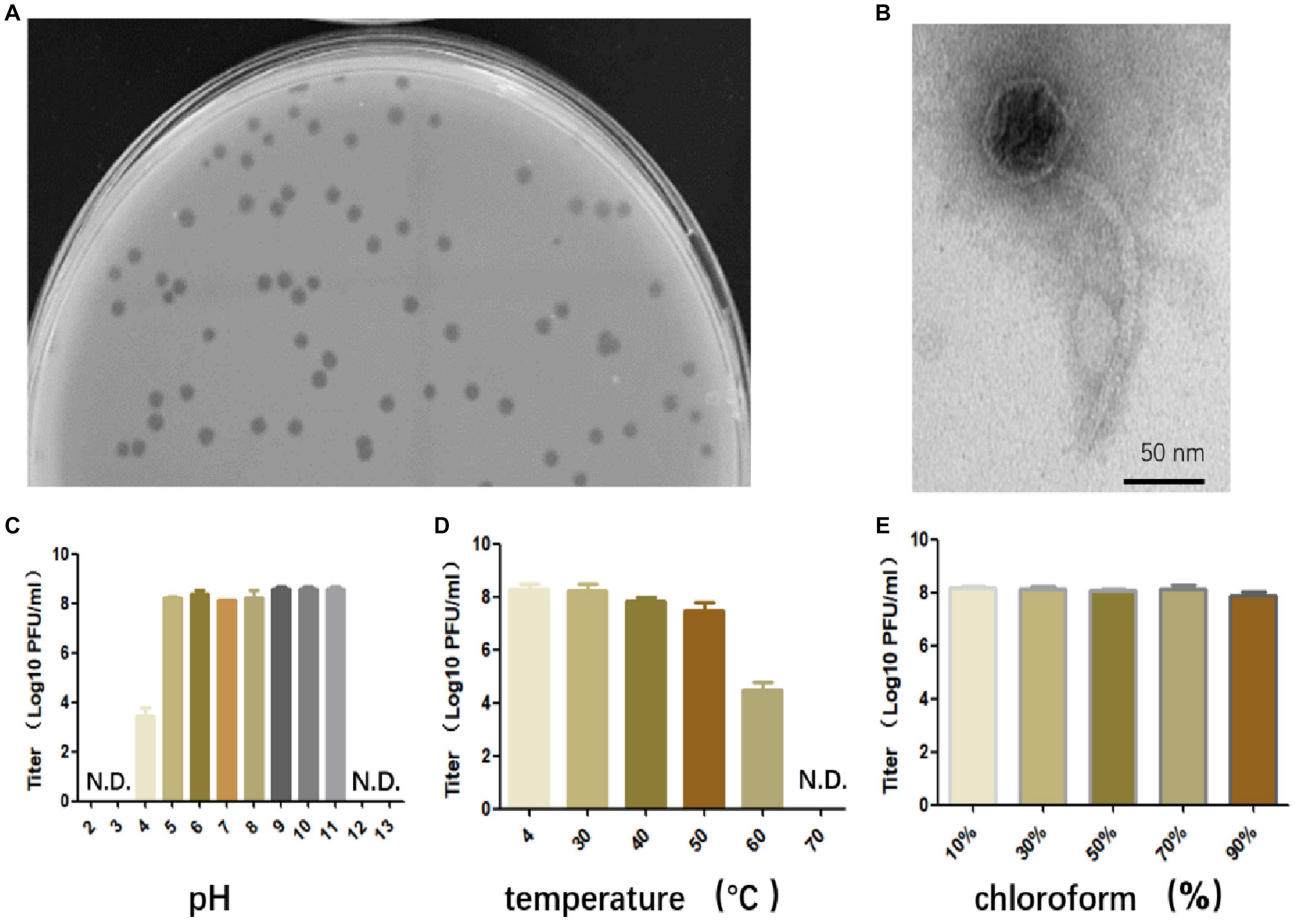
The biological characteristics of phage SFQ1. **(A)** The plaques of phage SFQ1 formed on a double-layered agar plate. **(B)** The transmission electron microscopy image displays the morphology of the phage SFQ1, with a scale bar of 50 nm. **(C)** Phage SFQ1 is stable over a broad range of pH values from 5 to 11. **(D)** SFQ1 could tolerate 50°C treatment but was completely inactivated at 70°C. **(E)** SFQ1 is completely resistant to chloroform treatment, and its titer is not changed for various concentrations of chloroform treatment.

Bacteriophage that specifically targets *E. faecalis* strain EFS01 was obtained from the sewage of the hospital. Phage plaques were first observed on a top agar lawn containing the clinical strain EFS01. The plaque was purified three times, resulting in the isolation of phage SFQ1. This phage formed clear plaques that were approximately 3 mm in diameter on a double-layered agar plate, as shown in Figure 1A. Electron microscopy revealed that phage SFQ1 had an icosahedral head with a diameter of around 50 nm and a long tail (Figure 1B). Thus, phage SFQ1 is a siphovirus and was designated vB_EFaS_SFQ1.

### Stability of bacteriophage

Phage SFQ1’s stability was evaluated under different pH, temperature, and chloroform treatments. The results revealed that SFQ1 remained viable within a pH range of 5 to 11 (Figure 1C). Additionally, SFQ1 was able to survive at 50°C but rapidly became inactivated at 60°C (Figure 1D). However, after chloroform treatment, the titer of SFQ1 is not changed using various concentrations of chloroform, indicating that it is completely resistant to chloroform (Figure 1E). These findings suggest that SFQ1 can withstand moderate acid and alkali conditions, high temperatures, and chloroform treatment.

### Biological characterization of bacteriophage

**Figure 2:**
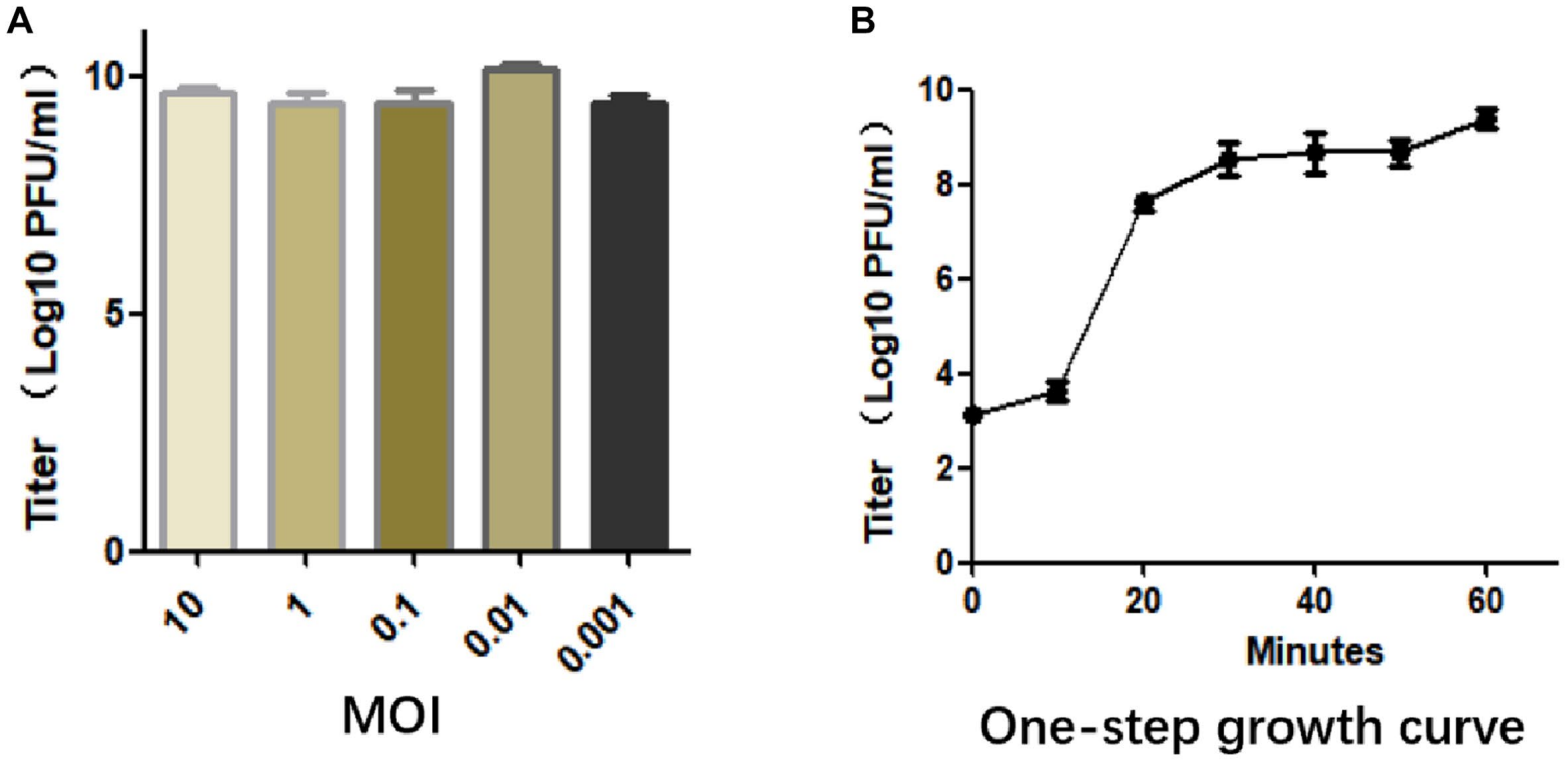
**(A)** The multiplicity of infection assay indicates that SFQ1 produced the most progenies when the phage and host bacteria were mixed at MOIs of 0.01. **(B)** The one-step growth curve for bacteriophage SFQ1 indicates that the phage titer increased after 10 min of infection and plateaued at 30 min, with a lysis time of approximately 30 min.

The optimal MOI was determined by mixing phage with bacteria at different ratios, and when the MOI was 0.01, it produced the maximum titer of particles (1.44 × 10^10^ PFU/mL), indicating that 0.01 is the optimal MOI of SFQ1 (Figure 2A).

To further characterized phage SFQ1, a one-step growth curve experiment was conducted, which showed that phage SFQ1 had a 10 min latent period and plateaued 30 min after phage infection (Figure 2B). Thus, the phage lysis time is about 30 min, and the burst size is about 110 PFU/cell based on the curve.

The host range of SFQ1 was estimated by plaque assays. Ten clinically isolated *E. faecalis* strains and five *E. faecium* strains were tested for their sensitivity against SFQ1. Six *E. faecalis* strains could be lysed by SFQ1, indicating a modest host range, while none of the *E. faecium* strains could be infected by SFQ1 (Table 1).

### Genomic characterization of phage SFQ1

**Figure 3:**
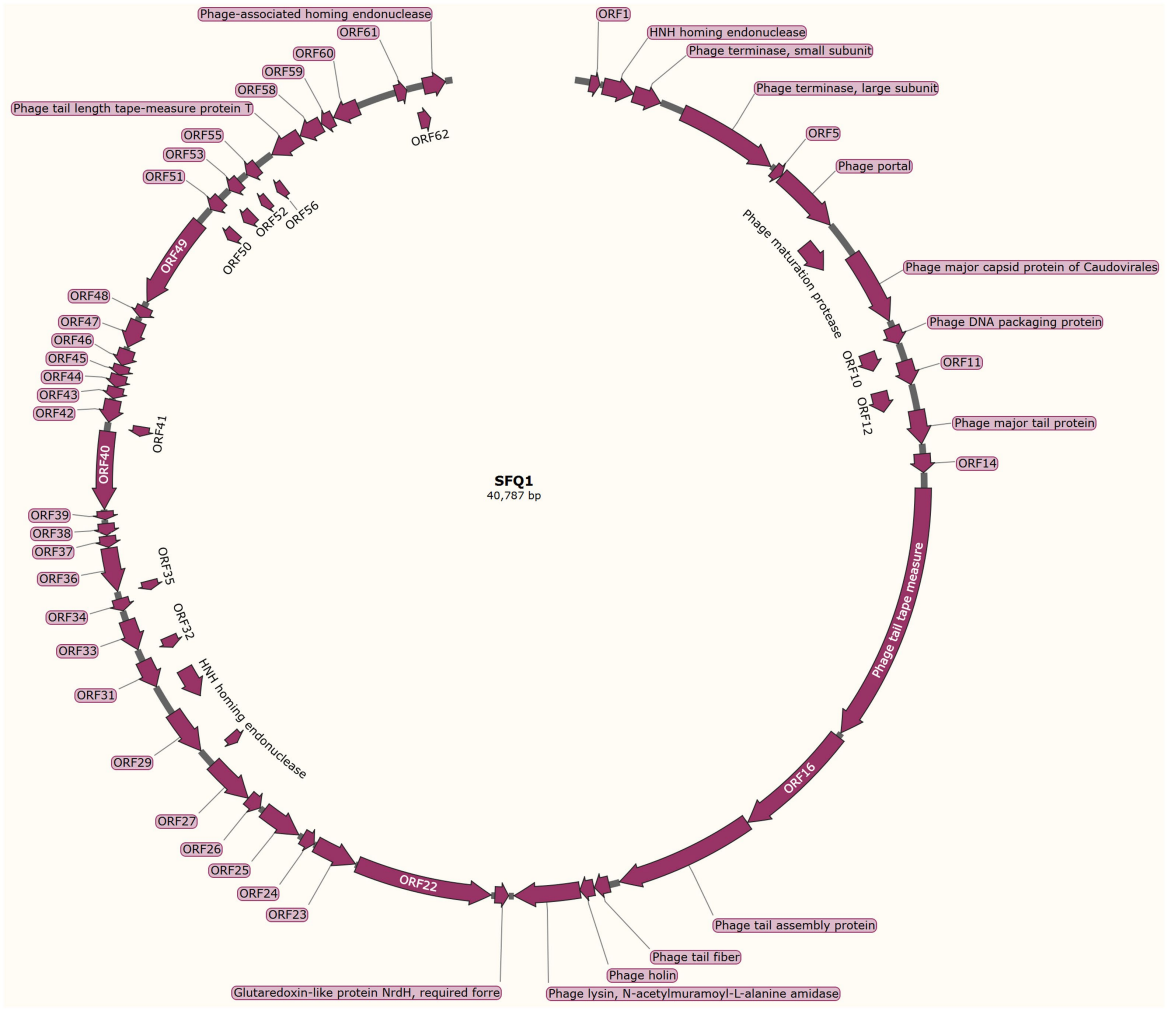
The genome characterization of phage SFQ1. The circular genome map of SFQ1 was generated using GraphPad Prism 8.0.2, and the annotation of ORFs was performed using RAST and the BLASTP database. The function of each predicted ORFs of phage SFQ1 are shown, and 17 out of the 63 predicted ORFs are functionally annotated.

The genome structure of SFQ1, a newly isolated *E. faecalis* phage, was investigated in this study. The phage genome was shown to be linear double-stranded DNA with a length of 40,787 base pairs and a G + C content of 35%. The genomic similarity of SFQ1 to *E. faecalis* Phage (NC_042126.1) was found to be 94.2%, based on sequence alignment. The SFQ1 genome encodes 63 predicted open reading frames (ORFs), with 17 of them considered functional genes and the remaining 46 annotated as hypothetical proteins (Figure 3). The complete genome sequence of SFQ1 is available at GenBank (accession no. OQ831052).

The annotated genes in SFQ1’s genome were genes involved in DNA replication and modification, phage genome packaging, structural proteins, and host lysis proteins modules. ORF2 was predicted to encode the HNH homing endonuclease, which is involved in phage DNA packing by cooperation with terminase, which is encoded by ORF3 and ORF4. While the phage structural proteins, such as tail fiber and capsid, are encoded in a specific region. ORF13 encodes the phage major tail protein, ORF15 encodes the phage tail tape measure protein, ORF17 encodes the phage tail assembly protein. The phage lysin, which includes a N-acetylmuramoyl-L-alanine amidase, is also predicted close to the tail fiber gene. But, most of the genes are unknown proteins, such as the genes that are used to control host metabolism are not annotated. And none of the ORFs encoded virulence factors or antibiotic resistance genes, and the genome of SFQ1 did not encode lysogenic modules, such as integrase or repressor proteins. These data indicate that SFQ1 is a strictly lytic phage and can be safely used to treat *E. faecalis* infections.

### Phylogenetic analysis

To better understand the evolution and relationship of phage SFQ1 with other *E. faecalis* phages, the genome of SFQ1 was compared with that of 20 *E. faecalis* phages, which were downloaded from NCBI that have homology with SFQ1.

**Figure 4:**
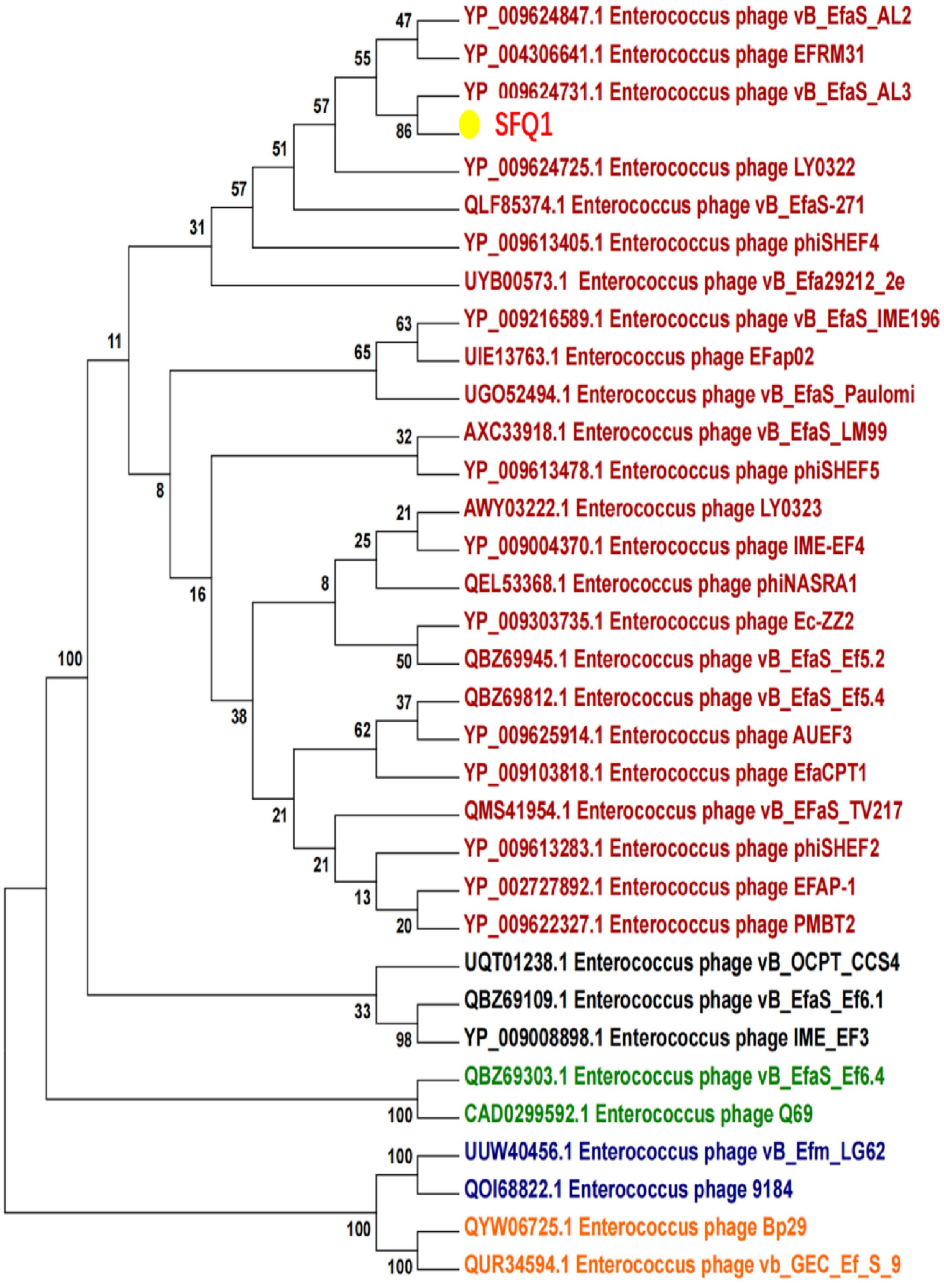
Phylogenetic analysis of SFQ1 and various *E. faecalis* bacteriophages based on the similarity of the whole genome sequences. SFQ1 was labeled in red in a clade with phage vB_EfaS_AL3.

**Figure 5:**
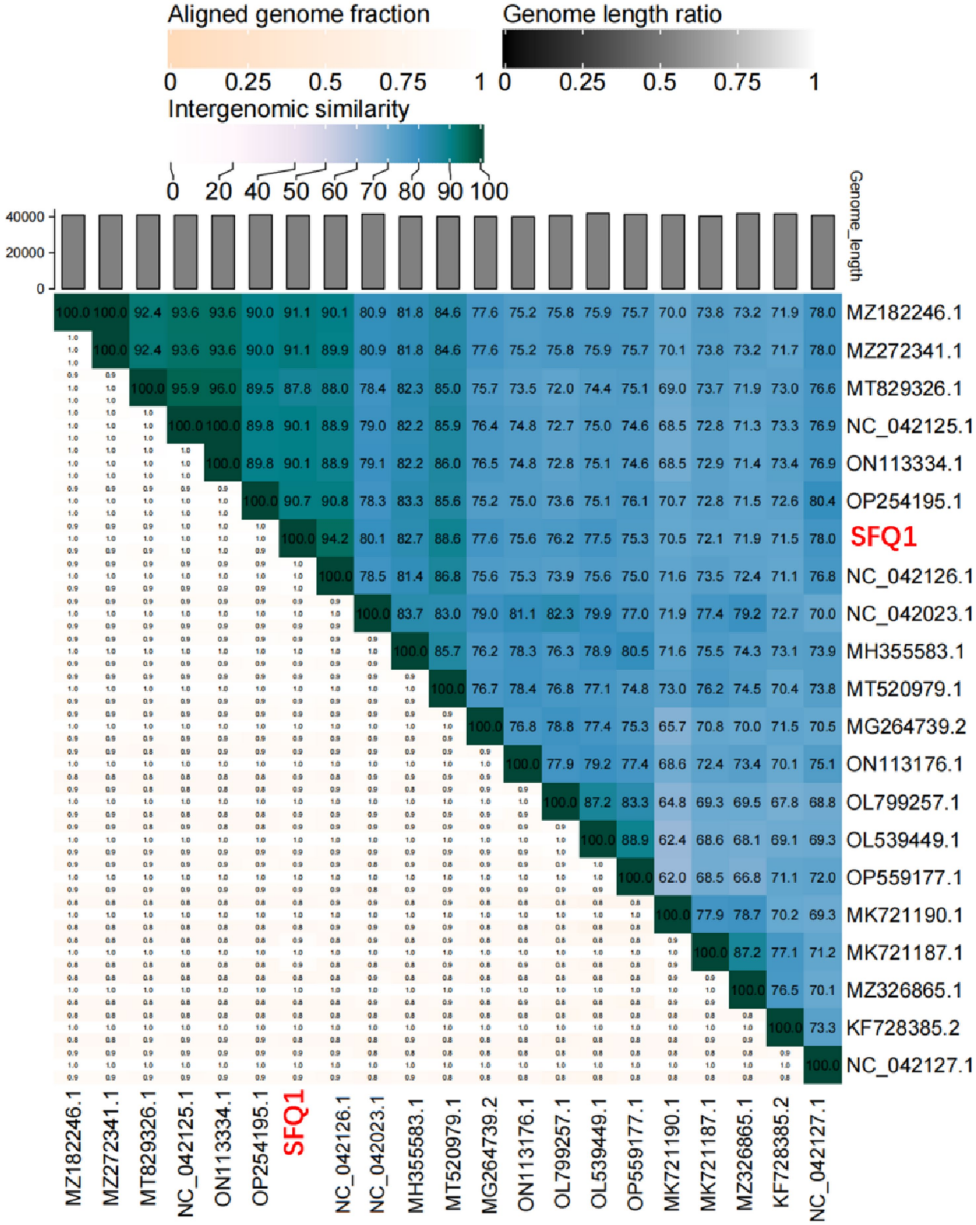
Heatmap of the average nucleotide identity values between SFQ1 and 20 most similar bacteriophages. According to a matrix of Hadamard values of pairwise alignment coverage and the percentage identity, the ANI values were generated and SFQ1 was marked in red.

Based on the whole genome sequences, the phylogenetic tree showed that IME278 was grouped into a clade with siphovirus, which is consistent with the results of the whole genome similarity analysis (Figure 4). Phage vB_EfaS_AL3 was highly similar to phage SFQ1. Then, we used VIRIDIC to calculate the intergenomic similarities, showing the similarity between phages SFQ1 and NC_042126.1 was 84.2%, while that with NC_042023.1 was 80.1% (Figure 5).

### Biofilm disruption

**Figure 6:**
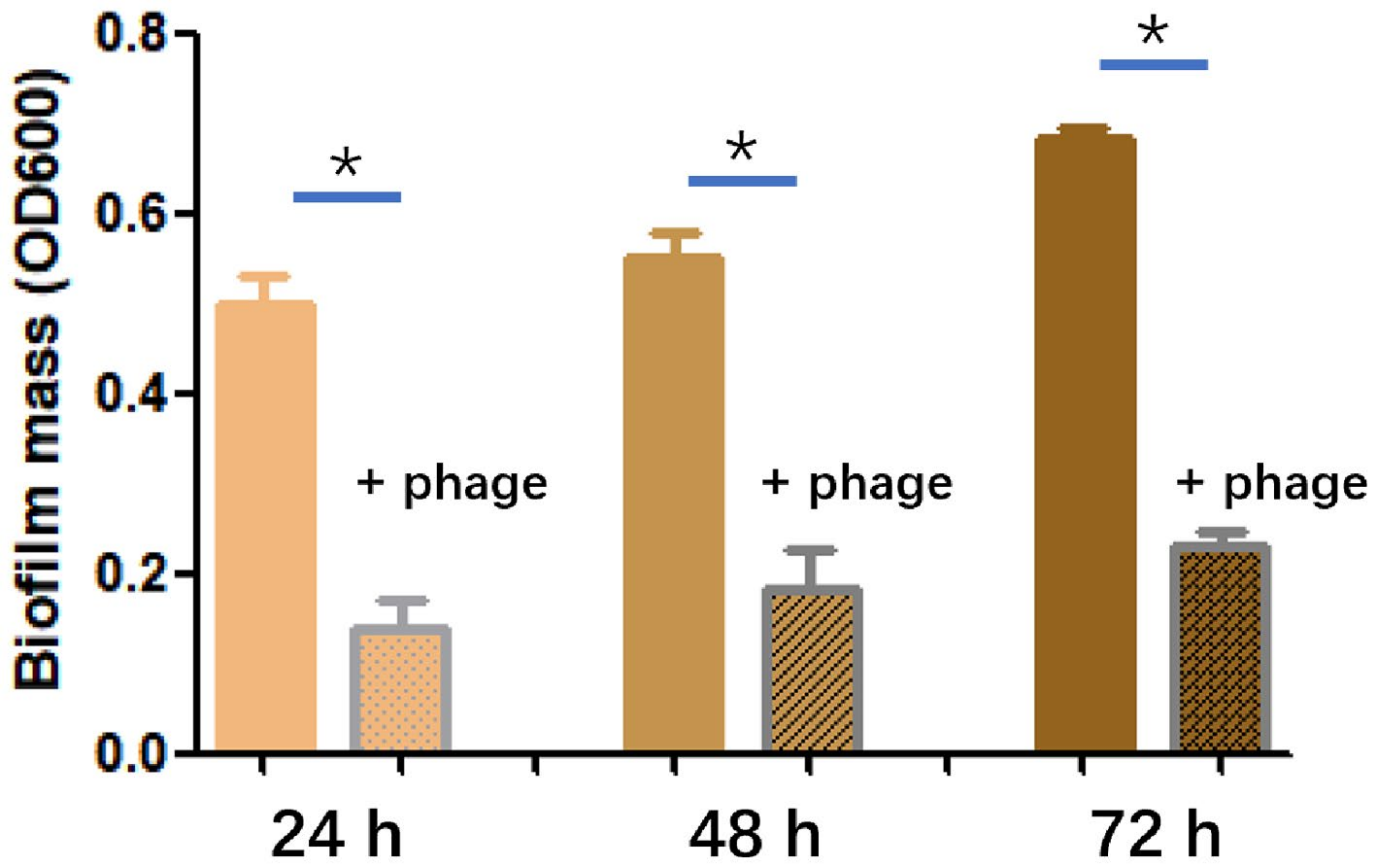
The effect of phage SFQ1 on biofilms formed by *E. faecalis* EFS01 was evaluated by measuring biofilm using crystal violet assay before and after phage treatment. Biofilms were formed for 24 h, 48 h, or 72 h, and were treated with phage SFQ1 for 6 h. The results showed that phage SFQ1 had a significant effect on biofilms formed for all three-time points. The asterisks indicate statistical significance (*p* < 0.05).

The efficiency of phage SFQ1 in destroying existing biofilms of *E. faecalis* EFS01 was assessed under various biofilm-forming conditions (Figure 6). Biofilm experiments were conducted thrice with duplicate readings, and the biofilm was grown to three-time points of 24 h, 48 h, and 72 h. At each stage, phage SFQ1 significantly reduced the *E. faecalis* EFS01 biofilm within 6 h, and the reduction was statistically significant (*p* < 0.05) (Figure 6), indicating that SFQ1 can disrupt the biofilm efficiently.

## Discussion

Phage therapy has been widely recognized as a promising strategy for treating infectious diseases caused by various bacteria, including *E. faecalis* (El Haddad et al., 2019). Thus, more and more *E. faecalis* phages were identified that targets different parts of the *E. faecalis* cell wall, and the cocktail with different phages could reduce the likelihood of phage resistance development (Banla et al., 2018; Al-Zubidi et al., 2019; Chatterjee et al., 2019). One study identified a cocktail of phages that were effective in killing antibiotic-resistant *E. faecalis* strains *in vitro* but not effective in the gut of mice (Buttimer et al., 2022). In addition to the well-known phage therapy, phages also play a vital role in treating many other diseases. For instance, (Duan et al., 2019) discovered phages that specifically target cytolysin-positive *E. faecalis* strains, which can cause hepatocyte lysis under the alcohol condition and lead to alcoholic hepatitis. The use of these phages has shown promising results in treating alcoholic hepatitis caused by *E. faecalis.* The development of phages with specificity against different *E. faecalis* strains has the potential to revolutionize the treatment of infectious diseases caused by this bacterium. Moreover, the characterization of *E. faecalis* phages can also shed light on their potential role in the gut microbiome and provide new therapeutic opportunities for gut microbiome-associated diseases. Therefore, further research and characterization of *E. faecalis* phages are essential for developing effective therapies against *E. faecalis* infections and other microbiome-associated diseases.

In this study, we characterized an effective *E. faecalis* phage SFQ1, it is a dsDNA phage with a large burst size, and lyse the host effectively. Moreover, its genome was sequenced and did not encode any lysogenic gene, antibiotic resistance gene, or virulent gene. Thus, SFQ1 is an effective and safe candidate for phage therapy.

Biofilms are communities of bacteria that can be highly resistant to antibiotics and can contribute to persistent infections (Khalifa et al., 2015; Sanz et al., 2017). Thus, researchers have investigated the potential of using phages to treat biofilm-related *E. faecalis* infections. A study found that a phage cocktail was effective in reducing *E. faecalis* biofilm formation *in vitro*, suggesting its potential use as a therapeutic agent (Khalifa et al., 2015). In this study, we also test the effect of SFQ1 on disrupting the existing biofilm, and we found that all three stage biofilms could be significantly disrupted by phage SFQ1, thus in clinical therapy, SFQ1 could be effective when combined with the use of antibiotics.

In conclusion, our findings highlight the potential of SFQ1 as a promising candidate for phage therapy against *E. faecalis* infections. Future studies should focus on identifying its specific receptor, which is critical for developing effective phage cocktails and minimizing the risk of phage resistance. Overall, this study suggested the importance of exploring the potential of phages as alternative therapies against antibiotic-resistant bacteria.

## Data availability statement

The datasets presented in this study can be found in online repositories. The names of the repository/repositories and accession number(s) can be found in the article/supplementary material.

## Author contributions

WG and XW conceived and designed the experiments. FS, JS, JT, and HX performed the experiments. FS and JS analyzed the data. WG and XW wrote the paper. All authors contributed to the article and approved the submitted version.

## Funding

This research was supported by Natural Science Foundation of Sichuan Province of China (2023NSFSC1902).

## Conflict of interest

The authors declare that the research was conducted in the absence of any commercial or financial relationships that could be construed as a potential conflict of interest.

## Publisher’s note

All claims expressed in this article are solely those of the authors and do not necessarily represent those of their affiliated organizations, or those of the publisher, the editors and the reviewers. Any product that may be evaluated in this article, or claim that may be made by its manufacturer, is not guaranteed or endorsed by the publisher.

## Footnotes

^1^ https://cge.cbs.dtu.dk/services/ResFinder/

^2^ https://cge.cbs.dtu.dk/services/VirulenceFinder/
